# Supporting the minority physician pipeline: providing global health experiences to undergraduate students in the United States–Mexico border region

**DOI:** 10.3402/meo.v20.27260

**Published:** 2015-06-17

**Authors:** Jose L. Burgos, Daniel Yee, Thomas Csordas, Adriana C. Vargas-Ojeda, Luis A. Segovia, Steffanie A. Strathdee, Jose A. Olivares-Nevarez, Victoria D. Ojeda

**Affiliations:** 1 Division of Global Public Health, School of Medicine, University of California San Diego, La Jolla, CA, USA; 2 Department of Anthropology, University of California San Diego, La Jolla, CA, USA; 3 Facultad de Medicina y Psicología, Universidad Autonoma de Baja California, Tijuana, Baja California, Mexico; 4 Prevencasa AC, Tijuana, Baja California, Mexico

**Keywords:** Mexico, undergraduate mentoring, minority physician pipeline, global health

## Abstract

**Background:**

The sizeable US Latino population calls for increasing the pipeline of minority and bilingual physicians who can provide culturally competent care. Currently, only 5.5% of US providers are Hispanic/Latino, compared with 16% of the US population (i.e., **>**50.5 million persons). By 2060, it is predicted that about one-third of all US residents will be of Latino ethnicity.

**Activities and outcomes:**

This article describes the Health Frontiers in Tijuana Undergraduate Internship Program (HFiT-UIP), a new quarterly undergraduate internship program based at a US–Mexico binational student-run free clinic and sponsored by the University of California, San Diego School of Medicine and the Universidad Autónoma de Baja California in Tijuana, Mexico. The HFiT-UIP provides learning opportunities for students and underrepresented minorities interested in medical careers, specifically Latino health.

**Discussion:**

The HFiT-UIP might serve as a model for other educational partnerships across the US–Mexico border region and may help minority and other undergraduates seeking academic and community-based enrichment experiences. The HFiT-UIP can also support students’ desires to learn about Latino, border, and global health within resource-limited settings.

The sizeable US Latino population calls for increasing the pipeline of minority and bilingual physicians who can provide culturally competent health care. This approach is recognized by the Institute of Medicine's (IOM) seminal report ‘Unequal Treatment: Confronting Racial and Ethnic Disparities in Healthcare’ (1, 2), which further suggests that a diverse provider workforce can lead to improvements in the patient–provider encounter (e.g., reduction in language barriers, perceived quality of care) (1, 3). Similar sentiments have been echoed by academic medicine faculty (4, 5) and to this end, the American Medical Association (AMA) and the Association of American Medical Colleges (AAMC) are promoting careers in medicine via programs targeting underrepresented minorities (e.g., the AMA ‘Physician Interview Project’, AAMC ‘Aspiring Docs’) (6, 7).

In 2008, only 5.5% of US providers were identified as Hispanic/Latino, compared with 16% of the US population (8). It is further predicted that by 2060, at least one-third of all US residents (or >128 million persons) will be of Latino ethnicity (9). Overall, 36% of Latinos are immigrants, resulting in a need for a bilingual healthcare workforce (10). Historically, Latinos have primarily settled in California, Florida, and Texas, where more than 50% of all US Latinos currently reside (11, 12). Yet, Latinos now live throughout the US, and the American South has experienced the greatest Latino population growth (13). The growth and geographic expansion of the Latino population means that Latino health is a national concern, necessitating concerted efforts to prepare a minority physician pipeline to address the lack of diversity in medical schools and to reduce the gaps in health disparities among the Latino population (1, 14).

This article describes the Health Frontiers in Tijuana Undergraduate Internship Program (HFiT-UIP), a new quarterly undergraduate internship based at a US–Mexico binational, student-run free clinic sponsored by the University of California, San Diego (UCSD) School of Medicine and the Universidad Autónoma de Baja California (UABC) in Tijuana, Mexico. Similar to global health experiences in medicine (15), this new program exposes underrepresented minorities and other undergraduate students to careers in medicine and related health professions. Students interested in Latino health also have opportunities to engage in culturally and linguistically appropriate care in a limited-resource setting that serves vulnerable populations. This program is consistent with the IOM's report “Who Will Keep the Public Healthy?” – which identifies cultural competence, ethics, community-based participatory research, and global health as major areas needing to be addressed in the coming years (2).

## HFiT clinic setting

In 2011, students from the UCSD and UABC Schools of Medicine – along with faculty from both universities – formed the HFiT clinic, the first binational student-run free clinic in the US–Mexico border region (16). Before 2011, professional relationships between faculty members from both institutions existed through collaborative cross-border research projects. However, faculty members identified a need among study participants for regular access to linguistically and cultural competent health care in Mexico. In response, they proposed a student-run free clinic, the HFiT clinic, which could not only provide care for the most disadvantaged persons in Tijuana but also serve as a training, research, and community service outpost.

The HFiT clinic is located in Tijuana's *Zona Norte* near the red light district and is situated less than a mile from the US–Mexico border and adjacent to the world's busiest international border crossing. The Zona Norte is a highly marginalized area in Tijuana, where drug use and sex work are pervasive, and hundreds of deported migrants and homeless persons live in the Tijuana River Canal's sewers. Culturally tailored health services are provided free of cost to uninsured, impoverished, and structurally vulnerable populations (16).

HFiT is supported by an interdisciplinary team of volunteers – including five physicians (two from UCSD and three from UABC), a volunteer nurse from the Baja California Health Department, a licensed clinical nutritionist from the Universidad de Estudios Avanzados in Mexico, two mental health counselors (one each from the University of San Diego [USD] and UABC), and a social worker from the Baja California Health Department. This multidisciplinary team ensures that patients are able to obtain a variety of care. The clinic has five exam rooms for primary care consultations (i.e., equipped with ophthalmoscopes, otoscopes, sphygmomanometers, and negatoscopes to view x-ray films), a minor surgical procedures room for treating abscesses, an ultrasound machine, a medical laser for tattoo removal, a dark-field/phase microscope connected to an LCD screen, and space for a walk-in mental health clinic. Additionally, there are two large classrooms for didactic activities.

Prevencasa, a Tijuana-based, non-profit grassroots organization, involved in harm reduction and HIV-prevention initiatives among marginalized populations in Tijuana's Zona Norte, provides the HFiT clinic with the physical space for the clinic to operate. US students, volunteers, and clinical faculty meet on the US side of the border and are provided two-way transportation between the border crossing and the HFiT clinic by a private van supported by the clinic and operated by Prevencasa. Other collaborators include the State of California Baja Health Department and USD.

The HFiT clinic provides educational, research, and community service opportunities to UCSD and UABC medical students in global health while offering free basic healthcare services (e.g., primary care and reproductive health services, social/community service referrals, health education, nutrition counseling, point-of-care glucose testing, and rapid testing for HIV and sexually transmitted infection) to structurally vulnerable populations (16). Between 30 and 40, disadvantaged patients receive primary healthcare services each Saturday.

In 2012, UCSD and UABC faculty saw a need to transition undergraduate HFiT clinic volunteers into unique roles via a program tailored to their specific needs and abilities. Before the development of the HFiT-UIP in 2013, no structured program existed for undergraduate students who volunteered at HFiT. It was determined that the clinic's administrative core would be improved by providing an infrastructure to facilitate undergraduates’ growing involvement in faculty-supervised activities. Furthermore, by creating an undergraduate program, the clinic would have a mechanism to provide credit for fieldwork hours completed for UCSD and UABC undergraduate students. It was envisioned that the new program could support undergraduate students passionate about careers in medicine, related health sciences (e.g., nursing), and/or global health.

In the fall of 2013, a HFiT-UIP pilot program accepted 12 undergraduates from those 23 students who had previously volunteered at clinic. The racial/ethnic breakdown of accepted undergraduate students was 20% white/Caucasian, 42% Hispanic/Latino, 31% Asian, and 4% Native American – which closely mirrored the larger applicant pool. Below, we describe the HFiT Undergraduate Internship Program in more detail.

## HFiT undergraduate internship program

### HFiT-UIP objectives

The HFiT-UIP provides a structured mechanism for UCSD and UABC undergraduates to learn about: 1) the provision of health care in a low-resource setting and 2) the healthcare needs of structurally vulnerable populations in the US–Mexico border region. Students also observe culturally competent care via interactions with clinical faculty from UCSD and UABC. The HFiT-UIP strives to provide underrepresented minorities and other undergraduate students with opportunities to become involved in Latino health care via professional development activities (e.g., shadowing providers, access to faculty and medical student mentors, leadership opportunities related to infrastructure development and sustainability of the clinic, and learning about the use of electronic medical records as a tool for patient care). The HFiT-UIP also provides Latino and other undergraduate students with a diverse range of role models in the health sciences and health professions (e.g., clinicians, researchers, clinician–researchers, administrators, and educators). Upon completion of the program, HFiT-UIP students will have: 1) gained knowledge of Latino health issues in the US–Mexico border region; 2) learned how to empathetically engage structurally vulnerable Latino populations; and 3) learned about healthcare delivery in an interdisciplinary, limited-resource clinical setting.

### HFiT-UIP content

The HFiT-UIP applies diverse teaching methods to promote student's learning, including classroom-based seminars, training in clinical activities (e.g., workshops on clinical procedures), and mentoring by senior students and faculty (Table 1). The HFiT-UIP exposes students to front-line public health issues affecting the border region, including barriers to health care, poverty, socioeconomic, and structural factors associated with providing care to vulnerable populations, and healthcare issues specific to population subgroups including substance users, injection drug users, sex workers, homeless persons, migrants, and deportees (i.e., migrants expelled from the US). Seminars include discussions on the influence of cross-border cultural and sociopolitical issues on the Latino population in the border region.

**Table 1:** Preclinical activities schedule: health frontiers in Tijuana Undergraduate Internship Program

Week	Topic	Teaching methods	Evaluation methods
1	Internship launch and overview Scheduling clinic visits Travel safety Student safety and travel requirements for Tijuana Mexico (check list) Question answer panel with (repeat) students Train students in use of electronic medical record, patient demographic survey, and use of devices to collect patient height, weight and blood pressure	Training at UCSD campus	Group discussion Timely and complete submission of travel documents
2	Tour of the HFiT clinic Confidentiality/safety issues Cultural competency Complete UCSD CITI ethics online training Ethics in global health education	On-site workshop at HFiT clinic in Tijuana Online ethics training On-site nurse-supervised workshop on vitals bureau	Group discussion Observation of students at vitals bureau by on-site nurse at HFiT clinic
3	Infection control guidelines and universal precautions Case studies	On-site workshop at HFiT clinic in Tijuana	Group discussion Observation of students at vitals bureau by on-site nurse at HFiT clinic
4	Mental health Biomedical and social aspects of substance abuse in the border regionFindings from ongoing research studies	On-site workshop at HFiT clinic in Tijuana	Group discussion Observation of students at vitals bureau by on-site nurse at HFiT clinic
5	Migration and health Deportation Circular migration	On-site workshop at HFiT clinic in Tijuana	Group discussion Observation of students at vitals bureau by on-site nurse at HFiT clinic
6	Overview of HIV and sexually transmitted infections in the border region	On-site workshop at HFiT clinic in Tijuana	Group discussion Observation of students at vitals bureau by on-site nurse at HFiT clinic
7	Sexually transmitted infections Case studies	On-site workshop at HFiT clinic in Tijuana	Group discussion Observation of students at vitals bureau by on-site nurse at HFiT clinic
8	Reproductive health	On-site workshop at HFiT clinic in Tijuana	Group discussion Observation of students at vitals bureau by on-site nurse at HFiT clinic
9	Epidemiology of Tuberculosis in the San Diego-Tijuana Region Case studies: TB outbreak in the General Hospital in Tijuana	On-site workshop at HFiT clinic in Tijuana	Group discussion Observation of students at vitals bureau by on-site nurse at HFiT clinic
10	Student as teacher Community health The community as teacher	On-site workshop at HFiT clinic in Tijuana	Group discussion Observation of students at vitals bureau by on-site nurse at HFiT clinic
Finals week	Wrap up/case studies/lessons learned/look to the future	On-site class at UCSD	Assessment of completion of hours, reflection of HFiT-UIP experience as an oral presentation Certificate provided for successful completion of internship

The HFiT-UIP incorporates several training opportunities for students – the first of which occurs at the beginning of the academic quarter. All students from Mexico and the US participate in an orientation session led by HFiT student leaders focusing on: 1) travel procedures; 2) safety in Mexico and at the clinic; 3) Spanish and English medical terminology; 4) border language slang; 5) HFiT's services; 6) HFiT's physical layout; 7) role playing; and 8) the registration system and demographic survey associated with patient intake and the electronic medical record system (EMS). Students are trained about patient confidentiality – and enroll in an online course pertaining to the Health Insurance Portability Accountability Act (HIPAA) training (or the Mexican equivalent), as well as the National Institutes of Health basic ethics training. Finally, students are introduced to procedures for collecting patient vital signs (i.e., height, weight, blood pressure, pulse, and temperature).

Once on-site at HFiT, students receive ongoing training and supervision as they undertake various administrative activities: 1) patient registration using the EMS (including administration of a brief demographic survey) and 2) taking patient vital signs under the supervision of a nurse or senior medical students. With patients’ permission, undergraduate students are able to shadow clinical encounters, and ‘interns’ are invited to participate in a journal club held in collaboration with medical students and faculty from UCSD and UABC medical schools. UCSD HFiT-UIP students are encouraged to attend selected lectures from MED239 – a preclinical elective that explores relevant border and public health issues. Their counterparts from UABC are invited to the *Fronteras Saludables* elective offered to UABC medical students. HFiT-UIP students participate in workshops and seminars to examine the roles of culture in the context of health-related beliefs and behaviors among marginalized individuals in the US–Mexico border region and develop skills to assess the significance of cultural factors in clinical practice. Thus, HFiT-UIP participants obtain didactic and practical field experience pertaining to medicine in a diverse Latino community.

### Eligibility for HFiT-UIP

UCSD undergraduate students enrolled in the Global Health program may satisfy the 100 h global health field experience by enrolling in two quarters of the HFiT-UIP. Students attend five Saturday full-day fieldwork sessions and receive 50 h of credit per quarter. UABC undergraduate premedical students can enroll in a four-unit semester course and are required to attend 10 Saturday full-day sessions. Both UCSD and UABC students can apply to the program up to two times per year via a standardized online application to participate in the UIP in their respective institutions. The selection committee composed of eight students and faculty evaluate applicants on the following domains: 1) whether applicants are from underrepresented minority groups, a first-generation college student, or from economically disadvantaged backgrounds; 2) prior experience in healthcare settings; 3) computer, language, and other skills (e.g., fundraising, web-design, writing); 4) sex; and 5) academic year with priority given to third and fourth year students who would benefit from the program to fulfill programmatic and graduation requirements. Grade point averages (GPA) are not used to exclude students, except in the cases of very low GPAs (e.g., 2.4), because involvement in the UIP may further detract from students’ ability to fulfill their academic program.

The HFiT-UIP is offered to UCSD students each quarter, including during the summer and winter breaks as the clinic operates year-round. For Mexican undergraduate students, the UABC academic program consists of semester-long (two-quarter) courses with one intersemester summer option for the months of July and August. UCSD students are not required to be fluent in Spanish as we provide opportunities to US undergraduate students to improve their Spanish language proficiency during their fieldwork rotations. Most of the HFiT faculty and UABC students are fully bilingual because the UABC School of Medicine requires premedical and medical students to be proficient in English language to access medical resources and peer-reviewed research publication (17).

### Evaluating HFiT-UIP interns

Because the HFiT-UIP is an experiential learning activity, no grades are assigned – though students receive ‘credit’ or ‘no credit’ for working in this mentored site. Students who do not fulfill the required number of hours per quarter do not receive a letter stating that they have completed the UIP; students can make up any sessions missed, if credit is requested. Students are also evaluated based on their attendance and performance in each of the UIP rotation stations (e.g., ability to register patients via the electronic medical record, ability to interact with HFiT medical team to assist with patient needs) and participation in didactic classroom meetings. At the end of the quarter, students are asked to give an oral presentation reflecting on their experiences in the UIP. The HFiT-UIP fieldwork experience can be used by UCSD Global Health majors to support the development of the required Capstone thesis project (not required for UABC undergraduate students).

### Challenges and next steps

The HFiT-UIP is limited to 24 students per quarter/semester from each institution, although demand exceeds the number of students that can be accommodated. Faculty members from the UCSD School of Medicine volunteer their time, but are also seeking to better integrate the program into the undergraduate campus. For UCSD students, participation is often impeded by logistical issues such as: 1) lack of transportation to the US–Mexico border; 2) parking costs and long wait times at the US border; and 3) lack of funding for obtaining a passport card or enrolling in a secure traveler program such as GLOBAL Entry or SENTRI (18) – which can expedite reentry into the US.

Although the growth of the HFiT-UIP is primarily related to funding, structured evaluations are needed to assess program objectives and the impact on students’ professional interests, aspirations, and career paths. Students have indicated that they rely heavily on their experiences at HFiT to support their graduate school applications and interviews, and thus far, two students have matriculated in medical school and one has entered a Masters in Social Work program. To further support learner involvement and growth, we are exploring the possibility of a limited number of longer term placements (i.e., three or more quarters) to HFiT-UIP students who have excelled by assuming leadership roles or assisting with specific projects – such as the creation of an online pharmacy/clinic equipment inventory or videos highlighting work at the HFiT clinic.

## Conclusion

The UCSD HFiT-UIP is a new undergraduate internship program designed to grow the pipeline of clinical providers interested in Latino health and capable of practicing responsive culturally and linguistically appropriate care. To our knowledge, this is the first binational, student-run free clinic in the US–Mexico border region that offers undergraduates academically and career-appropriate learning opportunities. Indeed, a full 90% of accepted undergraduate students are from minority groups (almost one-half from Hispanic or Latino communities), and it is hoped that the HFiT-UIP can continue to accommodate minority undergraduates seeking academic and community-based experiences that may nurture their transition to medical school and other health professions.
